# Comparison of Multispectral Image-Processing Methods for Brain Tissue Classification in BrainWeb Synthetic Data and Real MR Images

**DOI:** 10.1155/2021/9820145

**Published:** 2021-03-07

**Authors:** Hsian-Min Chen, Hung-Chieh Chen, Clayton Chi-Chang Chen, Yung-Chieh Chang, Yi-Ying Wu, Wen-Hsien Chen, Chiu-Chin Sung, Jyh-Wen Chai, San-Kan Lee

**Affiliations:** ^1^Center for QUantitative Imaging in Medicine (CQUIM), Department of Medical Research, Taichung Veterans General Hospital, Taichung, Taiwan; ^2^Department of Biomedical Engineering, Hungkuang University, Taichung, Taiwan; ^3^Department of Computer Science & Information Engineering, National United University, Miaoli, Taiwan; ^4^Department of Radiology, Taichung Veterans General Hospital, Taichung, Taiwan; ^5^School of Medicine, National Yang-Ming Chiao Tung University, Taipei, Taiwan; ^6^Department of Electrical Engineering, National Chung Hsing University, Taichung, Taiwan; ^7^Section of Radiology, College of Medicine, China Medical University, Taichung, Taiwan; ^8^Chief Strategy Officer, Tungs' Taichung MetroHarbor Hospital, Taichung, Taiwan

## Abstract

Accurate quantification of brain tissue is a fundamental and challenging task in neuroimaging. Over the past two decades, statistical parametric mapping (SPM) and FMRIB's Automated Segmentation Tool (FAST) have been widely used to estimate gray matter (GM) and white matter (WM) volumes. However, they cannot reliably estimate cerebrospinal fluid (CSF) volumes. To address this problem, we developed the TRIO algorithm (TRIOA), a new magnetic resonance (MR) multispectral classification method. SPM8, SPM12, FAST, and the TRIOA were evaluated using the BrainWeb database and real magnetic resonance imaging (MRI) data. In this paper, the MR brain images of 140 healthy volunteers (51.5 ± 15.8 y/o) were obtained using a whole-body 1.5 T MRI system (Aera, Siemens, Erlangen, Germany). Before classification, several preprocessing steps were performed, including skull stripping and motion and inhomogeneity correction. After extensive experimentation, the TRIOA was shown to be more effective than SPM and FAST. For real data, all test methods revealed that the participants aged 20–83 years exhibited an age-associated decline in GM and WM volume fractions. However, for CSF volume estimation, SPM8-s and SPM12-m both produced different results, which were also different compared with those obtained by FAST and the TRIOA. Furthermore, the TRIOA performed consistently better than both SPM and FAST for GM, WM, and CSF volume estimation. Compared with SPM and FAST, the proposed TRIOA showed more advantages by providing more accurate MR brain tissue classification and volume measurements, specifically in CSF volume estimation.

## 1. Introduction

Multispectral analysis techniques have been applied to the classification of brain magnetic resonance imaging (MRI) to more accurately differentiate between normal and diseased brain tissue [1–4]. In early publications, several methods involving *k*-means, fuzzy *c*-means, and *k*-nearest neighbors have been employed for the multispectral segmentation of magnetic resonance (MR) images [5–8]. However, those methods did not appear to be suitable for the robust segmentation of brain MRI, due to a number of limitations, such as uncertainty and vagueness of image data, requirement for long computation times, and high error rates in cerebrospinal fluid (CSF) segmentation [9, 10]. Recently, some software packages widely used in the scientific community have successfully implemented statistical and atlas-based techniques for brain MRI segmentation. These software packages include SPM, developed by the Wellcome Department of Imaging Neuroscience at the University College London (United Kingdom), and FMRIB's Automated Segmentation Tool (FAST), which is part of the FMRIB Software Library (FSL) and was implemented by the Analysis Group, FMRIB, Oxford (United Kingdom). The segmentation tools incorporated in these software packages are based on a mixture of the Gaussian mixture model (GMM) and a priori tissue probability maps (TPMs) with default parameters for yielding an accurate and consistent segmentation of a single MR image [11, 12]. In the recent versions of these software packages, multispectral segmentation has been extended to improve MRI segmentation. However, this method is highly dependent on knowledge of the distribution of the different tissue classes; furthermore, the quality of the implementation has not been tested, and much work remains in the area of validation.

Multispectral analysis techniques used in satellite imaging processing systems have been applied to tissue classification of brain MRI for decades because of the similarity between satellite and MR imaging data [13, 14]. However, these techniques are not practical for medical use because of the intense requirements on the relevant image-processing algorithms to extract the available information across the MR images. We previously developed a novel multispectral analysis method, derived from remote sensing techniques, for the robust classification of brain MRI. This method is implemented by applying the TRIO algorithm (TRIOA), which consists of an independent component analysis (ICA), a support vector machine (SVM), and an iterative version of Fisher's linear discriminant analysis [15]. The method combines the strengths of these three individual algorithms to facilitate the robust classification of brain tissue with the benefits of operating over a short computation time and in the native coordinate space, which avoids the registration problems that occur from the transformation to a standard coordinate space. With no requirement for probability maps to initiate classifications, the method proved effective for classifying gray matter (GM), white matter (WM), and CSF in normal young adults, healthy elderlies, and dementia patients [16].

In this paper, we investigated the accuracy of the TRIOA and the latest software package versions of the other classification tools of SPM and FAST by using a variety of synthetic normal brain data. For clinical MRI data with no available gold standard for in vivo experiment comparisons, the robustness of the classification tools was analysed using a quantitative volume assessment of GM, WM, and CSF in a series of normal adults with different ages.

## 2. Materials and Methods

### 2.1. Materials

#### 2.1.1. BrainWeb: Simulated Brain Database

This paper used the BrainWeb simulated database from the McConnell Brain Imaging Centre of the Montreal Neurological Institute, McGill University (http://www.bic.mni.mcgill.ca/brainweb), to assess the performance of the classification methods. The simulated normal MR images (181 × 217 × 181 voxel resolution) comprised T1-weighted imaging (T1WI), T2-weighted imaging (T2WI), and proton density imaging data with a 1 mm isotropic voxel size. Seven data sets of the synthetic image data were chosen with four noise levels of 0%, 1%, 3%, and 5%, and two intensity nonuniformity levels of 0% and 20%.

#### 2.1.2. Clinical Brain MRI

Clinical brain MRI data were acquired using a whole-body 1.5 T MRI system (Aera, Siemens, Erlangen, Germany) with a phase-array head coil. Three study groups were used, consisting of 35 young subjects (10 male, 25 female; 28.9 ± 5.9 years old), 54 middle-aged subjects (23 male, 31 female; 51.5 ± 5.6 years old), and 51 elderly subjects (29 male, 22 female; 66.9 ± 6.0 years old). The Institutional Review Board of Taichung Veterans General Hospital reviewed and approved the experimental protocol and the consent procedure (IRB numbers CE12233, CF14038, CG14039, and CE15021A). Written informed consent was obtained from all volunteers and patients. The imaging protocol involved three high-resolution 3-Dimensional Fourier Transformation (3DFT) acquisition sequences: T1WI, T2WI, and fast fluid-attenuated inversion-recovery (FLAIR). Other imaging parameters were a voxel size of 1 × 1 × 1 mm, a matrix of 256 × 256 × 176, and a number of excitation of 1.

#### 2.1.3. MR Data Preprocessing

The preprocessing of multispectral MR data in this paper included motion correction with rigid-body approach [17] to registering FLAIR and T2WI with T1WI, intensity inhomogeneity correction using N4ITK which was similar to the nonparametric nonuniformity intensity normalization (N3) method and better performance [18], and skull striping with FSL-brain extraction tool (BET) [19]. The default BET parameters were used with fractional intensity threshold was equal to 0.5 and threshold gradient was 0.

### 2.2. Evaluated Methods

#### 2.2.1. SPM

The “Unified Segment” version of SPM, or SPM8, is based on a parametric statistical model of the intensity patterns of MRI brain volume [11]. The theory of the parametric statistical model is adopted from the GMM, in which each tissue cluster is assumed to have a normal (Gaussian) distribution. The GMM is one of the most widely used approaches to solving the classification problem of MR images of brain tissue. This model includes only the intensity information, with no spatial information being considered. In a simple GMM, the probability function of the entire dataset *y* is derived by assuming that all brain tissue is independent and defined as follows:

(1) $$\begin{array}{c} P\left(y\left|\mu ,\sigma ,\gamma \right.\right)=\underset{\forall x\in L}{\sum }\gamma \cdot f\left(y\left|\theta_{x}\right.\right), \end{array}$$

where *x* is the random variable, and the model parameters *θ*_*x*_ = {*μ*_*x*_, *σ*_*x*_^2^} are the mean and variance of the Gaussian function. The mixing parameters *γ* can be included among the unknown parameters. A possible approach to solving the parameter estimation problem is to determine the maximum of the log-likelihood function. One of the most used methods of solving the maximization problem is to use the expectation-maximum (EM) algorithm, which can be used iteratively to update the mixture parameters. The TPMs are used as a priori information of the tissue classes. SPM8 uses four tissue classes, namely GM, WM, CSF, and nonbrain. However, histograms in a previous study show that the overlap between GM and WM is higher than 10% for a T1-weighted MR image [17]. Therefore, a segmentation method cannot satisfy the conditions of intensity distribution from one modality. One method of solving this problem is combining several modalities, such as T1-weighted, T2-weighted, or FLAIR, with different intensity contrasts that increase the reliability of tissue segmentation and reduce the reliance on observer input.

SPM8 New Segment is an extension of the default Unified Segment. The algorithm in New Segment is essentially the same as that described in [11], with two crucial changes. First, the New Segment method provides the ability to use multispectral MR image data to classify brain tissue. Second, in the New Segment version of SPM8, six different TPMs are used, namely GM, WM, CSF, bone, soft tissue, and background. The background class primarily includes air and other nonbrain tissue. The other slight modifications from the Unified Segment version of SPM8 are different registration and deformation parameters.

The latest version of SPM is SPM12, which was released on October 1, 2014. The unified segmentation section of SPM8 is replaced by a modified version of New Segment in SPM12. The theory of segmentation in SPM12 is the same as that in SPM8 New Segment. Its implementation, which is based on the algorithm presented in [11], enables multispectral classification and incorporates a more flexible image registration component. The changes of SPM12 compared with New Segment include different regularization for the deformations, some different default settings, and the reintroduction of the rescaling of the TPMs.

In addition, the TPMs were regenerated using the T2-weighted and proton density-weighted scans from the IXI dataset [18]. Unless otherwise specified, the default parameters of SPM8, SPM8 New Segment, and SPM12 in this paper were used for multispectral classification. The probabilities of classifying brain tissue in SPM were thresholded at 0.5 for computing the similarity index [19], which is widely used for evaluating classification algorithms.

#### 2.2.2. FSL-FAST

FAST is based on a hidden Markov random field (HMRF) model and an associated EM algorithm. The principle of the HMRF model originates from the hidden Markov model (HMM). The original HMM was designed as a one-dimensional Markov model; however, it cannot solve 2D or greater problems such as image segmentation. Accordingly, FAST is used as a special case of an HMM in which the underlying stochastic process is a Markov random field instead of the HMRF model. Mathematically, a HMRF model is characterized by the inclusion of a hidden random field, observable random field, and conditional independence. Accordingly, a HMRF model with a Gaussian emission distribution can be specified as

(2) $$\begin{array}{c} p\left(y_{i}\left|x_{N_{i}},\theta \right.\right)=\underset{l\in L}{\sum }f\left(y_{i};\theta_{l}\right)p\left(l\left|x_{N_{i}}\right.\right), \end{array}$$

where *f*(*y*_*i*_; *θ*_*l*_) is the emission probability function and the parameter set *θ* = {*θ*_*l*_, *l* ∈ *L*} and *x*_*N*_*i*__ is a neighborhood configuration. Therefore, the parameter set *θ* = {*θ*_*l*_, *l* ∈ *L*} must be solved. The FAST procedure for estimating the parameter set *θ* = {*θ*_*l*_, *l* ∈ *L*} employs the EM algorithm. More specifically, the FAST method seeks an EM solution for three dependent unknowns: the bias field, the image classification, and the model parameters. A more detailed description of FAST is provided in [12].

#### 2.2.3. Our Purposed Method—TRIOA

We developed TRIOA, a novel multispectral analysis method for the robust classification of brain MRI [15, 16, 20, 21]. The TRIOA method consists of an ICA, an SVM, and Fisher's linear discriminant analysis (FLDA). ICA uses data sphering to remove data samples from the first and second statistics of the MR image data to facilitate separating different brain tissue structures in a set of statistically independent components. The method has been proved effective for enhancing the image contrasts of GM, WM, and CSF, which served as a preprocessing method for further brain classification [22]. Specifically, the ICA substantially contributes to accurate classification of SVM without employing optimal parameters or specified kernels. The SVM, a classification-based discriminant function, was originally developed to solve the classification problem on the basis of statistical learning theory [23]. Its major strength is that the required training data samples can be relatively small, which is beneficial in reducing the large-scale learning task. Minimizing the training samples can effectively reduce human intervention and the operating burden for manually labelling the target tissues. FLDA is widely used in statistics, pattern recognition, and machine learning to determine a linear combination of features for the characterization or classification of different subjects or events [24]. However, as a powerful supervised classifier, FLDA requires a sufficiently large pool of training samples to reflect the global properties of the class distributions in order to produce reliable classification. Generally, such a method may suffer from large measurement variability and difficulty getting accurate class labels of many training data. To resolve this problem, the SVM classifier is considered a preprocessing technique for FLDA to provide a larger pool of training data with enough brain tissue properties to initiate an iterative version of FLDA that can yield a consistent classification.

## 3. Results

### 3.1. Evaluation of BrainWeb Database

The CSF, GM, and WM tissues displayed in the MR images were classified using seven levels (n0rf0, n1rf0, n3rf0, n5rf0, n1rf20, n3rf20, and n5rf20).  Table 1 presents the similarity index (SI) [16] values for the CSF, GM, and WM tissues displayed in the T1WI, T2WI, and PDWI MR images that were estimated using the multispectral imaging methods of TRIOA, SPM12, SPM8-New Segment, and FAST (which were, respectively, defined in this paper as “TRIOA,” “SPM12-m,” “SPM8-N-m,” and “FAST-m”) to compare the classification results of these tissues with their corresponding ground truth.  Table 2 shows the SI values for the CSF, GM, and WM tissues displayed in the T1WI MR images that were estimated using the single-image classification methods of SPM12, SPM8, SPM8-New Segment, and FAST (which were, respectively, defined in this paper as “SPM12-s,” “SPM8-s,” “SPM8-N-s,” and “FAST-s”). Furthermore, a comparison of SI values derived using all the multispectral imaging and single-image classification methods revealed that TRIOA was the most effective at classifying GM and CSF tissues. FAST-m failed to accurately detect GM, WM, and CSF tissues at the n0rf0 level; thus, the SI values for those tissues were not obtained.

### 3.2. Evaluation of Clinical Brain MRI

**Figure 1:**
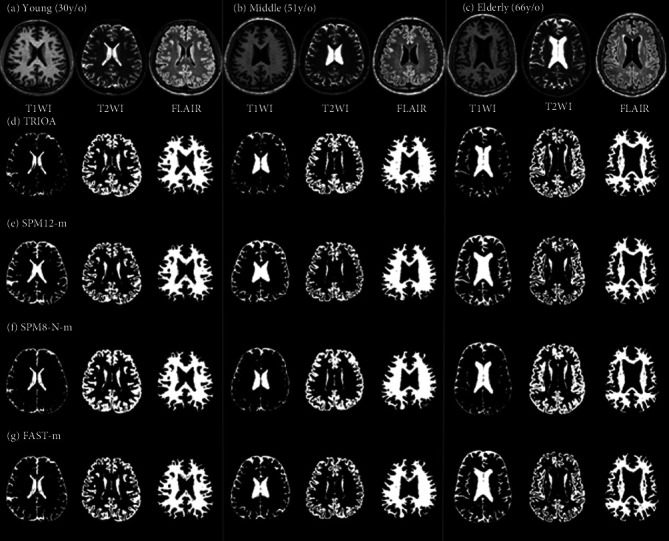
Classification of the CSF, GM, and WM tissues by performing TRIOA, SPM8-N-m, SPM12-m, and FAST-m on the brain images of three age groups. (a) T1WI, T2WI, and FLAIR MR images of young adults (30 years old); (b) T1WI, T2WI, and FLAIR MR images of middle-aged adults (51 years old); (c) T1WI, T2WI, and FLAIR MR images of elderly adults (66 years old); (d) results obtained using TRIOA on the original MR images presented in each group; (e) results obtained using SPM12-m on the original MR images presented in each group; (f) results obtained using SPM8-N-m on the original MR images presented in each group; and (g) results obtained using FAST-m on the original MR images presented in each group.

**Figure 2:**
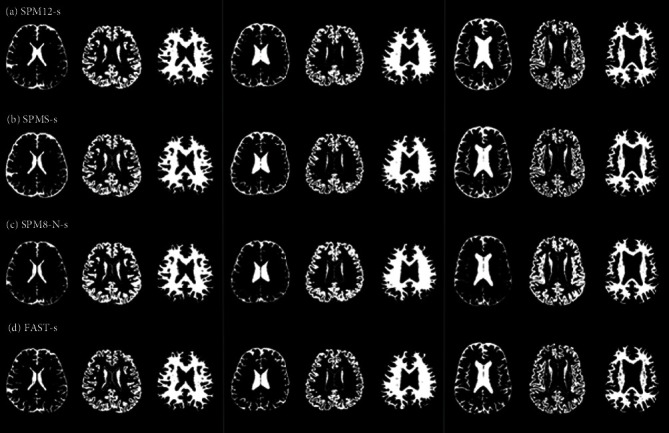
Classification of CSF, GM, and WM tissues by performing SPM12-s, SPM8-s, SPM8-N-s, and FAST-s on the brain images of three age groups. (a) Results obtained using SPM12-s on the original MR images presented in each group of Figure 1; (b) results obtained using SPM8-s on the original MR images presented in each group of Figure 1; (c) results obtained using SPM8-N-s on the original MR images presented in each group of Figure 1; and (d) results obtained using FAST-s on the original MR images presented in each group of Figure 1.

The real brain MR images of 140 subjects were also used to classify major tissues and evaluate the performance of all the methods. Subsequently, the four multispectral imaging methods (TRIOA, SPM12-m, SPM8-N-m, and FAST-m) were performed on the T1, T2, and FLAIR MR images.  Figure 1 shows the classification results for CSF, GM, and WM tissues obtained using the methods on the brain images of subjects in three age groups. The single-image classification methods of SPM12-s, SPM8-s, SPM8-N-s, and FAST-s were also performed to classify the CSF, GM, and WM tissues in the same brain images (Figure 2).

Table 3 lists the average volume fractions of the CSF, GM, and WM in the MR images of 140 subjects included in three groups of young, middle-aged, and elderly adults. These volume fractions were estimated using the multispectral classification methods of TRIOA, SPM8-N-m, SPM12-m, and FAST-m on the images to classify the three brain major tissues.  Table 4 presents the average volume fractions of the CSF, GM, and WM in the same MR images, which were estimated using the single-image classification methods of SPM12-s, SPM8-s, SPM8-N-s, and FAST-s on the images.

**Figure 3:**
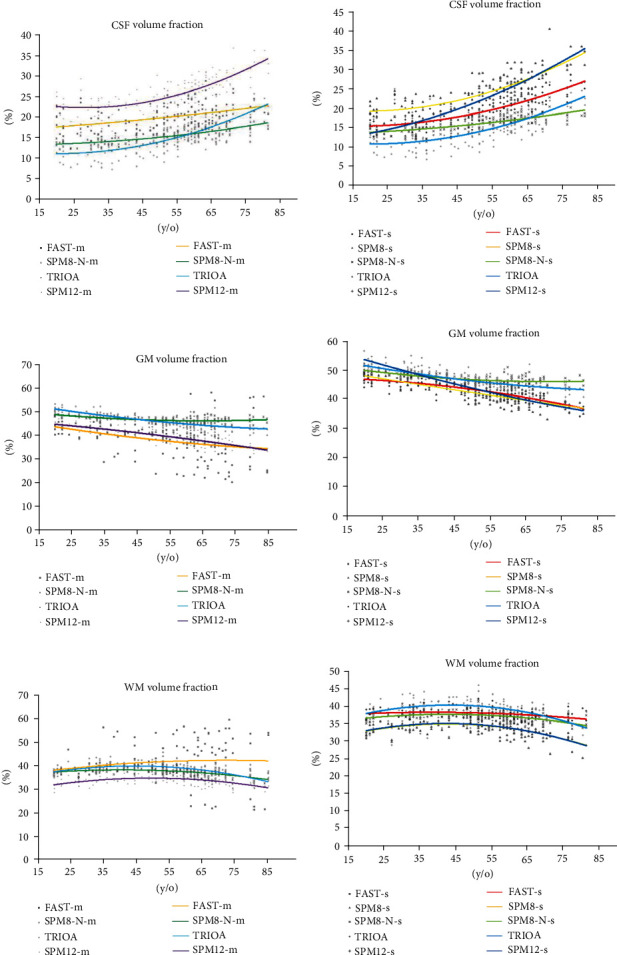
Relationships between age of subject and the average volume fractions of CSF, GM, and WM displayed in all the sample MR images. (a), (c), and (e), respectively, show the average volume fractions of CSF, GM, and WM estimated using TRIOA, SPM12-m, SPM8-N-m, and FAST-m; whereas (b), (d), and (f), respectively, show the average volume fractions of CSF, GM, and WM estimated using TRIOA, SPM8-s, SPM8-N-s, SPM12-s, and FAST-s.

Figure 3 presents the relationships between age and the average volume fractions of the CSF, GM, and WM displayed in all MR images of 140 subjects. These relationships are presented in the form of curves plotted by estimating the second-order polynomial regression equation. Figures 3(a), 3(c), and 3(e), respectively, show the average volume fractions of CSF, GM, and WM estimated using TRIOA (red), SPM12-m (dark blue), SPM8-N-m (dark green), and FAST-m (dark yellow); whereas Figures 3(b), 3(d), and 3(f), respectively, show the average volume fractions of CSF, GM, and WM estimated using TRIOA (red), SPM8-s (light yellow), SPM8-N-s (light green), SPM12-s (dark blue), and FAST-s (light blue).

### 3.3. CSF

The CSF volume in the brain of an adult is approximately 150 mL [25]. CSF typically accounts for 7-12% of the brain volume of an adult and increases to 16-25% in old age [26]. As Tables 3 and 4 show, only the average CSF volume fractions of young adults estimated using TRIOA and SPM8-N-m confirmed the findings of [25, 26]; those estimated using FAST-s, SPM8-N-s, and SPM12-s supported the findings of the relevant literature; and those estimated using FAST-m, SPM12-m, and SPM8-s were considerably higher than values reported in past studies. Further, the average CSF volume fractions estimated with all the classification methods increased with subject age, as indicated by Figures 3(a) and 3(b). Notably, the results in both figures showed that the average CSF volume fractions estimated using SPM8-s and SPM12-m differed markedly from those estimated using the other classification methods. The average CSF volume fractions estimated using both methods were higher by over 140 mL compared with those estimated using the other methods, with the average CSF volume fraction in elderly subjects being more than 28% higher (Tables 3 and 4).

Figure 1 (a) shows that CSF does not account for the entire lateral ventricles of young adults in the T1WI, T2WI, and FLAIR images. However, as the classification results of Figures 1(d)–1(g) and Figures 2(a)–2(d) show, only TRIOA, SPM8-N-m, SPM12-m, and FAST-m detected tissues other than CSF. Notably, TRIOA detected the largest number of non-CSF regions, whereas the other three single-image classification methods categorized lateral ventricle tissues as CSF. In young, middle-aged, and elderly subjects, all the single-image classification methods yielded higher CSF levels near the cranium than did the multispectral imaging classification methods.

### 3.4. GM

By using least-squares regression as a predictive model, Ge et al. [27] determined a linear decline of 45%–55% in the average GM volume fraction across the span of early to late adulthood (the subjects in their study were aged 20–86 years). Courchesne et al. [26] showed that GM volume grew by 13% between early (19–33 months old) to late (6–9 years old) childhood and, after 9 years of age, declined linearly at a rate of approximately 5% per decade throughout life. In addition, they observed no substantial difference in the average GM volume fraction between males and females (F: 58.5% ± 0.08%; M: 59.9% ± 0.07%).

Consistent with the studies, the current study determined that the average GM volume fractions estimated using all the classification methods decreased with age, as indicated by Tables 3 and 4 and Figures 3(c) and 3(d). Tables 3 and 4 reveal discrepancies in the average GM volume fraction between young and elderly subjects: 64.8 mL (5.0%) when determined using TRIOA, 16.0 mL (1.6%) when determined using SPM8-N-m, 78.9 mL (6.4%) when determined using SPM12-m, 66.2 mL (5.6%) when determined using FAST-m, 90.6 mL (7.0%) when determined using SPM12-s, 94.4 mL (6.6%) when determined using SPM8-s, 30.9 mL (2.4%) when determined using SPM8-N-s, and 72.9 mL (5.8%) when determined using FAST-s.

Based on the findings, SPM12-s and SPM8-s yielded an age-related difference of over 6.6% for GM volume, whereas SPM8-N-m produced the smallest difference (1.6%). However, only TRIOA supported the findings of [27, 28]. Lüders et al. [28] estimated the ranges of GM volume in 50 young male adults (49.4%–58.49%; mean: 54.51%) and 50 young female adults (52.12%–59.62%; mean: 55.71%), whereas Ge et al. [27] observed a 4.9% difference in the average GM volume fraction between young and elderly adults. The current study also determined that GM volume decreased with subject age across all the eight classification methods, as observed in Figures 3(c) and 3(d). Both figures show that the average GM volume fractions estimated using SPM8-N-s and SPM8-N-m trended steadily downward.

### 3.5. WM

WM volume typically grows by 74% between early childhood and adolescence, after which it increases slowly until the age of 40 years and decreases thereafter [26, 27]. Our findings showed that the WM volume fraction increased with age across almost all the classification methods, peaked at approximately the age of 45, and trended downward thereafter (Tables 3 and 4; Figures 3(e) and 3(f)). However, WM volume fractions estimated using FAST-m increased slightly after the age of 45 (Figure 3(e)) and were higher than GM volume fractions for middle-aged and elderly groups (Table 3), which contradicted the findings of previous studies.

## 4. Discussion

### 4.1. Influence of Different Classification Methods on CSF Readings

Lüders et al. [28] estimated the average GM volume fractions for young male (*n* = 50; 25.1 ± 4.5 years old) and female (*n* = 50; 24.2 ± 4.2 years old) adults; the average GM volume fraction for males was 270 mL (17.85%), whereas that for females was 230 mL (17.15%). Callaert et al. [29] reported that the average CSF fraction was larger in their elderly subjects (*n* = 18; 9 males and 9 females; 66.2 ± 3.4 years old) than in their young subjects (n = 21; 8 males and 13 females; 23 ± 1.7 years old), and the age effects were more pronounced when estimated using SPM than with all of the other methods (*p* < 0.0003), because the CSF estimate for the elderly group (479 ± 78.0 mL) determined by SPM was far larger than those determined by the other methods.

Many of the previous studies on GM, CSF, and WM tissue classification have provided limited discussion on the classification results regarding CSF tissues, because such results vary widely across different methods. In this investigation, it was determined that, across different methods, the average CSF volume fraction was 11.3%–22.2% for the young group, 14.4%–24.4% for the middle-aged group, and 16.7%–28.7% for the elderly group, with a difference of at least 11% in the CSF volume fraction range for each group (Tables 3 and 4). Callaert et al. [29] also reported inconsistent CSF estimates in the same groups obtained using different methods.

The accuracy of CSF classification can affect not only whole-brain volume estimation but also the estimation of GM and WM volumes. For example, Valverde et al. [30] suggested that considering the differences in sulcal cerebrospinal fluid can directly affect the accuracy of GM tissue classification. The main factor affecting the accuracy of CSF classification pertains to the characteristics of SPM and FAST. Based on voxel intensity histograms, both classification methods are typically performed on T1 MPRAGE images, in which the distribution of CSF gray-level intensity is similar to that of other background noises, increasing the difficulty of using the methods to accurately classify CSF tissues from the images. However, newly developed versions of SPM (e.g., SPM8 New Segment and SPM12), which are designed to classify T2-weighted and FLAIR images, have demonstrated improved classification results. Such improvement is evidenced by the findings of [31], who obtained more accurate estimates of intracranial volume (ICV) by using T2-weighted images than by using T1-weighted images.

### 4.2. Effects of Spatial Normalization and Parameter Adjustment on Classification Results

Brain tissue classification methods are used to quantitatively measure changes in the cerebral structure. We applied the proposed TRIOA, as well as software tools (SPM8, SPM12, and FSL-FAST) that are commonly used in neuroscientific research as multispectral imaging and single-image classification methods, to compare the classification results among different age groups. Across all of the classification methods used in this paper, higher CSF volume fractions and lower WM volume fractions were observed in the elderly group than in the young group, correlating strongly with the findings of previous studies [26, 27, 29, 32]. However, we determined that the age-related increases in the GM volume fraction for all the age groups when using SPM12-m differed the most from those obtained using the other methods, because the GM estimates determined by SPM12 were the lowest.

The aforementioned finding supports the results of [29], who examined the effects of classification methods and spatial normalization procedures on age-related GM reduction, and of [31], who proposed an SPM-based method to improve the accuracy of ICV measurement. Callaert et al. [29] observed that SPM tended to overestimate age-specific variations of GM volume, whereas DARTEL based on SPM8 New Segment tended to underestimate these structural changes. Hansen et al. [31] concluded that multispectral classification can lead to a slight underestimation of ICV.

Two possible explanations for the differences of readings across classification methods are proposed. First, newer classification methods use a larger number of tissue probability maps (TPMs). Compared with the TPMs of SPM8 and older versions, which detect only GM, WM, and CSF tissues, those of SPM8 New Segment and the most recently released SPM12 detect additional tissue categories including soft tissues, skull, and out-of-brain regions. Thus, such newer methods should theoretically be more accurate at assigning voxels to appropriate tissue categories [29, 33, 34].

The second explanation involves parameter adjustment. We applied the ICBM space template of East Asian brains and the corresponding default parameters for MR image classification.  Table 5 tabulates the default parameters for SPM8, SPM8 New Segment, and SPM12.

The number of terms for warping regularization of SPM increased from one to five when the software was updated from version 8 to 12. This algorithmic enhancement is demonstrated in the Appendix of the article of [33], which constitutes a more sophisticated regularization model of SPM12 that comprises five penalty terms (absolute displacement, membrane energy, bending energy, linear elasticity, and divergence). The purpose of this algorithmic enhancement is to improve SPM classification accuracy, as confirmed by [33], who used SPM8 New Segment, SPM12, and FreeSurfer to estimate the ICV volume of the T1 MPRAGE images of 288 subjects and then compared the ICV measurement results of the three automated methods with those of manual (standard) classification. Subsequently, they observed a strong correlation between SPM12 ICV and manual ICV (*R*^2^ = 0.940%) and reported that the correlation with manual measurements for SPM12 was significantly higher than for SPM8 and FreeSurfer. However, in this study, we compared the accuracy of the three methods only based on the total ICV estimates, providing no comparison of the GM, WM, and CSF classification results between these methods.

Peelle et al. [34] used voxel-based morphometry (VBM) to adjust for global effects and measure age-related GM reduction in healthy elderly adults. The authors also proposed a research direction for studying how the selections of TPMs, classification processes, and templates affect VBM results. Callaert et al. [29] compared the methodological differences in normalization between SPM5/SPM8 and DARTEL and suggested that DARTEL yields more accurate anatomical results, because it uses the SPM8 New Segment, creates a custom template [35], and performs an iterative process to progressively refine warping parameters for the transformation from individual subject spaces to a common space. Furthermore, the DARTEL procedure is more accurate than the standard SPM5/SPM8 procedure, because the latter uses a young-adult template and causes classification inaccuracies involving age-related effects. Buckner et al. [36] identified the construction and use of the target atlas as a major limitation of atlas-based normalization; they used a young-adult template to classify older-adult images, but this approach resulted in multiple failures.

In contrast to the above methods, TRIOA has two strengths. First, TRIOA classifies cerebral tissues within an individual native space. Conversely, SPM8, SPM12, and FAST classify using a template to warp image data from individual native spaces to a common space and convert them back to native spaces after classification; however, both conversions cause classification inaccuracies. Second, TRIOA eliminates the requirement for parameter settings. Whereas support vector machine (SVM) classification with the radial basis function kernel requires the specification of the penalty parameters *C* and *γ*, SVM with independent component analysis converts data attributes into the numerical form to reduce the complexity of numerical estimation and the effects of parameter optimization [16, 20, 37]. Thus, this method can be applied to reduce the complexity of numerical estimation and the effects of parameter optimization for MR image classification, thereby improving the accuracy of the measurement of brain tissue volume.

### 4.3. Potential Advantages of the TRIOA Method as an Emerging Artificial Intelligence (AI) Technique

In recent years, as the processing speed of computer hardware has increased substantially, many artificial intelligence (AI) technologies have been realized. There are many literatures that apply deep learning methods for brain tissue or brain tumor segmentation on MR images [38, 39]. Havaei et al. [40] proposed a deep neural network (DNN) for brain tumor segmentation on MRI, mainly to improve the traditional convolutional neural network (CNN) calculation method and increase the computed speed by at least 30 times. McClure et al. [41] proposed a Bayesian DNN method for brain tissue segmentation, and the results also showed that it can solve the current uncertainty problem of segmentation. In addition, Yang et al. [42] also proposed the deep CNN methods for neonatal brain tissue segmentation on MRI and explored the versatility of the two architectures of LiviaNET and HyperDense-Net. The experimental results found that the performance of HyperDense-Net architecture for neonatal brain tissue segmentation was better than LiviaNET. However, these deep learning technologies will face a very important challenging—that is, limited training and ground truth data [38]. The ground truth data requires manual operations by physicians, which is generally not feasible in clinical applications. To address this issue, the TRIOA is designed to be used as the labeling preprocessing of AI technology for brain tissue classification to reduce the manual operation required for physicians and further improve the feasibility of AI technologies in medical image-processing application.

## 5. Conclusions

In this paper, we propose a multispectral MR image classification method, TRIOA, which was applied in the analysis of real brain MR images of 140 subjects, who were categorized into three age groups: young, middle-aged, and elderly adults. We also compared the classification results with those of SPM8, SPM8 New Segment, SPM12, and FAST. Age-related differences in the GM and WM volumes ascertained using TRIOA were the most like those reported in previous studies; notably, the CSF volumes were relatively closer to the reasonable range. In addition, a comparison of multispectral imaging and single-image classification methods showed that, whereas SPM and FAST entailed parameter adjustment and the selection of spatially normalized templates, TRIOA eliminated these two requirements, thereby facilitating the accurate cross-sectional comparison of brain tissue volume among different age groups.

## Figures and Tables

**Table 1:** SI values for CSF, GM, and WM tissues in T1WI, T2WI, and PDW MR images using TRIOA, SPM12-m, SPM8-N-m, and FAST-m to compare the classification results of these tissues with the ground truth from BrainWeb.

Similarity index	TRIOA			SPM12-m			SPM8-N-m			FAST-m		
	GM	WM	CSF	GM	WM	CSF	GM	WM	CSF	GM	WM	CSF
n0rf0	**0.977**	**0.986**	**0.961**	0.460	0.610	0.423	0.738	0.869	0.398	—	—	—
n1rf0	**0.971**	**0.979**	**0.958**	0.769	0.895	0.628	0.865	0.923	0.646	0.329	0.694	0.551
n3rf0	**0.951**	0.958	**0.952**	0.892	0.956	0.753	0.935	0.958	0.814	0.899	**0.967**	0.716
n5rf0	**0.932**	0.939	**0.948**	0.906	0.950	0.797	0.929	0.948	0.823	0.844	0.921	0.693
n1rf20	**0.963**	0.972	**0.954**	0.796	0.921	0.630	0.865	0.923	0.641	0.890	**0.976**	0.708
n3rf20	**0.950**	0.957	**0.951**	0.894	0.957	0.755	0.925	0.957	0.776	0.898	**0.968**	0.716
n5rf20	**0.931**	0.938	**0.947**	0.908	0.950	0.798	0.930	0.950	0.817	0.768	0.869	0.693

**Table 2:** SI values for CSF, GM, and WM tissues in T1WI MR images using SPM12-s, SPM8-s, SPM8-N-s, and FAST-s to compare the classification results of these tissues with the ground truth from BrainWeb.

Similarity index	SPM12-s			SPM8-s			SPM8-N-s			FAST-s		
	GM	WM	CSF	GM	WM	CSF	GM	WM	CSF	GM	WM	CSF
n0rf0	0.795	0.614	0.839	0.892	0.879	0.839	0.949	0.961	0.851	0.818	0.807	0.769
n1rf0	0.905	0.901	0.848	0.936	0.958	0.846	0.947	0.963	0.853	0.865	0.901	0.761
n3rf0	0.925	0.952	0.821	0.934	0.960	0.853	0.938	0.958	0.846	0.900	0.962	0.739
n5rf0	0.917	0.940	0.824	0.922	0.939	0.807	0.918	0.936	0.831	0.887	**0.951**	0.729
n1rf20	0.906	0.902	0.848	0.934	0.958	0.845	0.943	0.961	0.846	0.879	0.925	0.764
n3rf20	0.927	0.954	0.824	0.932	0.959	0.850	0.939	0.958	0.845	0.903	0.963	0.749
n5rf20	0.918	0.941	0.823	0.921	0.940	0.851	0.920	0.937	0.831	0.893	**0.953**	0.749

**Table 3:** Average volume fractions of the CSF, GM, and WM displayed in the 140 MR images of typical young, middle-aged, and elderly adults that were estimated using TRIOA, SPM8-N-m, SPM12-m, and FAST-m.

Methods	Tissues	Young (20-39 y)		Middle-aged (40-59 y)		Old (>60 y)	
		(mL)	(%)	(mL)	(%)	(mL)	(%)
TRIOA	CSF	144.6 ± 41.6	11.3 ± 2.7	188.9 ± 46.0	14.4 ± 2.9	233.1 ± 54.9	18.2 ± 3.2
	GM	630.7 ± 73.2	49.7 ± 1.8	596.5 ± 51.1	45.8 ± 2.0	565.9 ± 52.1	44.7 ± 2.5
	WM	493.5 ± 62.0	38.9 ± 2.6	518.1 ± 56.4	39.7 ± 2.2	472.1 ± 61.4	37.1 ± 2.4
SPM8-N-m	CSF	177.7 ± 28.1	13.7 ± 1.2	199.1 ± 27.6	15.1 ± 1.5	219.0 ± 39.1	16.7 ± 1.8
	GM	622.2 ± 71.5	48.2 ± 0.9	617.8 ± 52.3	46.8 ± 1.0	606.2 ± 59.2	46.6 ± 1.5
	WM	491.6 ± 56.7	38.1 ± 1.2	504.0 ± 47.6	38.1 ± 1.2	476.7 ± 56.1	36.6 ± 1.5
SPM12-m	CSF	285.5 ± 52.5	22.2 ± 2.9	321.2 ± 45.1	24.4 ± 2.6	372.5 ± 62.0	28.7 ± 3.0
	GM	566.7 ± 68.7	44.1 ± 2.2	532.5 ± 48.8	40.6 ± 2.2	487.8 ± 51.8	37.7 ± 2.4
	WM	434.5 ± 55.3	33.8 ± 2.0	460.8 ± 50.2	35.0 ± 1.6	436.5 ± 57.6	33.6 ± 2.0
FAST-m	CSF	228.8 ± 53.5	17.8 ± 3.3	258.3 ± 44.8	19.7 ± 2.8	276.1 ± 56.5	21.3 ± 2.7
	GM	539.7 ± 81.1	42.2 ± 3.8	511.1 ± 79.2	39.0 ± 4.7	473.5 ± 139.3	36.6 ± 9.6
	WM	510.0 ± 73.2	40.0 ± 4.7	539.1 ± 77.7	41.2 ± 5.2	539.9 ± 133.4	42.1 ± 10.1

**Table 4:** Average volume fractions of the CSF, GM, and WM displayed in the 140 MR images of typical young, middle-aged, and elderly adults that were estimated using SPM12-s, SPM8-s, SPM8-N-s, and FAST-s.

Methods	Tissues	Young (20-39 y)		Middle-aged (40-59 y)		Old (>60 y)	
		(mL)	(%)	(mL)	(%)	(mL)	(%)
SPM12-s	CSF	203.6 ± 58.1	15.5 ± 3.7	258.6 ± 57.9	19.2 ± 3.6	314.7 ± 63.7	23.8 ± 3.7
	GM	661.0 ± 80.5	50.5 ± 2.5	613.8 ± 52.7	45.8 ± 2.7	570.4 ± 56.1	43.5 ± 2.7
	WM	445.3 ± 56.9	34.0 ± 2.4	469.9 ± 52.6	35.0 ± 2.1	430.9 ± 57.1	32.7 ± 2.3
SPM8-s	CSF	272.3 ± 49.0	19.7 ± 2.7	336.7 ± 61.7	23.7 ± 3.4	387.3 ± 65.3	28.2 ± 3.6
	GM	639.8 ± 72.7	46.4 ± 1.7	590.6 ± 51.4	41.8 ± 2.6	545.4 ± 58.4	39.8 ± 2.7
	WM	467.6 ± 58.7	33.9 ± 2.2	487.4 ± 53.5	34.4 ± 1.9	441.3 ± 60.2	32.1 ± 2.3
SPM8-N-s	CSF	188.9 ± 30.7	14.4 ± 1.5	219.0 ± 31.7	16.3 ± 1.5	234.1 ± 35.4	17.8 ± 1.6
	GM	636.3 ± 72.6	48.6 ± 0.9	624.1 ± 52.7	46.4 ± 1.2	605.4 ± 54.0	46.2 ± 1.4
	WM	483.7 ± 57.2	37.0 ± 1.5	501.7 ± 50.4	37.3 ± 1.2	472.0 ± 53.5	36.0 ± 1.3
FAST-s	CSF	205.5 ± 45.3	16.0 ± 2.7	251.9 ± 46.2	19.1 ± 2.7	291.5 ± 52.7	22.7 ± 2.7
	GM	590.1 ± 66.1	46.2 ± 1.8	562.9 ± 45.6	42.9 ± 1.8	517.2 ± 54.9	40.4 ± 2.3
	WM	483.0 ± 59.8	37.8 ± 1.6	499.0 ± 53.4	38.0 ± 1.7	473.2 ± 52.5	36.9 ± 1.5

**Table 5:** Default parameters for SPM8, SPM8 New Segment, and SPM12.

Parameter	SPM8	SPM8 New Segment	SPM12
MRF parameter	—	—	1
Clean up	Do not do clean up	—	Light clean
Warping regularization	1	4	(0, 0.001, 0.5, 0.05, 0.2)

“–” denotes N/A.

## Data Availability

Answer: Yes. Comment: The data used to support the findings of this study are included within the article.
